# Economic and Environmental Impact of Digital Health App Video Consultations in Follow-up Care for Patients in Orthopedic and Trauma Surgery in Germany: Randomized Controlled Trial

**DOI:** 10.2196/42839

**Published:** 2022-11-24

**Authors:** Jennifer Muschol, Martin Heinrich, Christian Heiss, Alher Mauricio Hernandez, Gero Knapp, Holger Repp, Henning Schneider, Ulrich Thormann, Johanna Uhlar, Kai Unzeitig, Christian Gissel

**Affiliations:** 1 Department of Health Economics Justus Liebig University Giessen Giessen Germany; 2 Department of Trauma, Hand and Reconstructive Surgery University Hospital Giessen Giessen Germany; 3 Bioinstrumentation and Clinical Engineering Research Group Bioengineering Department, Engineering Faculty Universidad de Antioquia Medellín Colombia; 4 Institute of Medical Informatics Justus Liebig University Giessen Giessen Germany

**Keywords:** carbon neutrality, digital health, environmental impact, health economics, net-zero, orthopedic, sustainability, telemedicine, trauma surgery, video consultations

## Abstract

**Background:**

Following the Riyadh Declaration, digital health technologies were prioritized in many countries to address the challenges of the COVID-19 pandemic. Digital health apps for telemedicine and video consultations help reduce potential disease spread in routine health care, including follow-up care in orthopedic and trauma surgery. In addition to the satisfaction, efficiency, and safety of telemedicine, its economic and environmental effects are highly relevant to decision makers, particularly for the goal of reaching carbon neutrality of health care systems.

**Objective:**

This study aims to provide the first comprehensive health economic and environmental analysis of video consultations in follow-up care after knee and shoulder interventions in an orthopedic and trauma surgery department of a German university hospital. The analysis is conducted from a societal perspective. We analyze both economic and environmental impacts of video consultations, taking into account the goal of carbon neutrality for the German health care system by 2030.

**Methods:**

We conducted a prospective randomized controlled trial comparing follow-up care with digital health app video consultations (intervention group) to conventional face-to-face consultations in the clinic (control group). Economic impact included the analysis of travel and time costs and production losses. Examination of the environmental impact comprised the emissions of greenhouse gases, carbon monoxide, volatile hydrocarbons, nitrogen oxides, and particulates, and the calculation of environmental costs. Sensitivity analysis included calculations with a higher cost per ton of carbon dioxide equivalent, which gives equal weight to the welfare of present and future generations.

**Results:**

Data from 52 patients indicated that, from the patients’ point of view, telemedicine helped reduce travel costs, time costs, and production losses, resulting in mean cost savings of €76.52 per video consultation. In addition, emissions of 11.248 kg of greenhouse gases, 0.070 kg of carbon monoxide, 0.011 kg of volatile hydrocarbons, 0.028 kg of nitrogen oxides, and 0.0004 kg of particulates could be saved per patient through avoided travel. This resulted in savings of environmental costs between €3.73 and €9.53 per patient.

**Conclusions:**

We presented the first comprehensive analysis of economic and environmental effects of telemedicine in the follow-up care of patients in orthopedic and trauma surgery in Germany. Video consultations were found to reduce the environmental footprint of follow-up care; saved travel costs, travel time, and time costs for patients; and helped to lower production losses. Our findings can support the decision-making on the use of digital health during and beyond the COVID-19 pandemic, providing decision makers with data for both economic and environmental effects. Thanks to the pragmatic design of our study, our findings can be applied to a wide range of clinical contexts and potential digital health applications that substitute outpatient hospital visits with video consultations.

**Trial Registration:**

German Clinical Trials Register DRKS00023445; https://tinyurl.com/4pcvhz4n

## Introduction

Medical care does not always require patients’ attendance in the hospital [1], mainly because digital health affords physicians and patients the opportunity to have synchronous video consultations online [2]. When used for outpatient follow-up care in orthopedic and trauma surgery, for example, video consultations can relieve patients of any restrictions on their mobility or of the need to travel long distances [3-5]. Patient satisfaction, physician satisfaction, and clinical outcomes often show comparable results between telemedicine and conventional face-to-face (F2F) examinations in the hospital, demonstrating that video consultations can be a safe and efficient alternative for patient care in orthopedic and trauma surgery [6-13].

After the outbreak of the COVID-19 pandemic, the role of digital health has been highlighted by the Riyadh Declaration [14]. Following the global pandemic response, there has been an increasing interest in telemedicine in clinical practice to reduce potential disease spread as well as in science, which is reflected in a growing number of literature reviews [2,15-20]. The number of clinical trials, however, remains limited. In particular, there are only a few health economic analyses of the use of telemedicine in orthopedic and trauma surgery follow-up care [15,21].

In addition to patient satisfaction and quality of care, the societal perspective needs to consider both economic and environmental effects in order to support stakeholders in deciding whether to implement telemedicine in orthopedic and trauma surgery. Following the United Nations Sustainable Development Goals, the 125th German Medical Assembly declared in 2021 that the German health care system should become carbon-neutral by 2030 [22]. One way of meeting this requirement might be the implementation of video consultations to supplement or substitute clinic consultations. Whether this is possible, however, must first be determined by investigations. A positive environmental impact of telemedicine has already been demonstrated in certain cases: for example, in the reduction of carbon monoxide, carbon dioxide, and nitric oxides [23-25]. However, analyses of the environmental impact of video consultations in the field of orthopedic and trauma surgery are limited, and no studies based on German data exist to date.

The aim of this study is to provide the first health economic analysis comparing telemedicine in the follow-up of patients in orthopedic and trauma surgery with knee and shoulder disorders with conventional F2F examinations in the clinic in Germany. The analysis focuses on the societal perspective, considering, on the one hand, the patients’ point of view in terms of potential time and cost savings and, on the other hand, the environmental impact regarding potential savings of emissions and environmental costs.

## Methods

### Study Design

The data used for the health economic analysis were obtained by a prospective randomized controlled trial (RCT) conducted at a single German university hospital—University Hospital Giessen, Department of Trauma, Hand and Reconstructive Surgery, Level-1 trauma center—between September 2020 and April 2021. The RCT was reported according to the Consolidated Standards of Reporting Trials (CONSORT) [26]. Patients in orthopedic and trauma surgery were randomly assigned 1 to 1 to an intervention group or a control group for a single follow-up appointment. The intervention group did not attend a standard outpatient follow-up appointment in the clinic but had a real-time online video consultation with the treating physician instead. The control group, on the other hand, was treated conventionally and received a F2F examination in the clinic. In both the intervention group and the control group, the examinations were performed by the same physicians. The study population had already received conservative or surgical treatment for various knee and shoulder conditions in the clinic.

### Ethical Considerations

Patients who were eligible for the study based on the inclusion and exclusion criteria in Textbox 1 were asked either at the clinic or by telephone if they wished to participate in the RCT. After a detailed verbal explanation of the study, including the conduct of a health economic analysis as part of the study, all study participants provided written informed consent. To protect the privacy of participating patients, pseudonymization of the study data took place. Study participants were not compensated for their participation. The local ethics committee of the University of Giessen approved the RCT (AZ 73/20), and the study was registered in the German Clinical Trials Register (DRKS00023445).

**Inclusion and exclusion criteria of the randomized controlled trial.**

**Inclusion criteria:**

- 18 years or older
- Previous outpatient or inpatient stay at the clinic, with an operation or conservative therapy
- Need of a follow-up that does not require more than a visual examination
- Ownership of a computer, laptop, tablet, or smartphone with microphone and camera
- Stable internet connection
- Mental and physical ability to consent and to participate
- Sufficient knowledge of German in order to understand the declaration of consent
- Shoulder International Classification of Diseases, Tenth Revision (ICD-10) codes: M75.1, M75.6, M75.0, Z96.60, M75.4, M19.91, S43.1, S42.20, S42.00, M75.2, M75.3, and S43.0
- Knee ICD-10 codes: S83.53, S83.54, S83.2, S83.0, M22.0, M23.32, M23.35, M17.1, M17.5, M21.16, M21.06, S83.3, S83.44, S83.43, S82.18, S82.0, S72.3, S72.43, M25.56, M76.5, S83.6, S76.1, and S86.8

**Exclusion criteria:**

- Neurological diseases that preclude the use of digital devices
- Diagnosis of dementia, blindness, or deafness
- Need for presence in the clinic and on-site treatment and diagnostics (ie, imaging, laboratory, stitches, and drainage)
- Appointments where the patient has to be touched and moved by the treating physicians
- Lack of willingness to participate
- Failure to consent

### Sample Size and Randomization

The sample size calculation of the underlying RCT was based on an a priori power analysis. As a conservative estimate, we used half of the effect size of 2.19 that was observed for the findings of patient satisfaction with telemedicine in a study by Sharareh and Schwarzkopf [8]. The effect size of 1.095 yielded 19 patients per study arm for a power of 90% in a 2-sided *t* test with a 5% significance level. To increase statistical power and to compensate for potential withdrawals and dropouts, missing responses, and a skewed distribution of results, the number of participants was expanded to 30 patients for each group. In total, 60 eligible patients were recruited for the study.

Using block randomization with randomly varying block sizes (ie, 4, 6, and 8), 30 patients were assigned to a follow-up with telemedicine (intervention group), and 30 patients were assigned to a conventional F2F follow-up in the clinic (control group). The parallel-design randomization and assignment process was performed independently of the treating physicians by study staff using sealed envelopes.

### Course of the Study

The video consultations in the intervention group were browser based for physicians and multiplatform for patients, including a digital health app or browser-based software from a German telemedicine provider. The software complies with the legal requirements in Germany and is recognized by the National Association of Statutory Health Insurance Physicians. The university hospital paid a monthly fee for each physician to use the software. Video consultation procedures were deliberately kept as simple and as functional as possible to ensure that they would be viable in regular clinical practice: all video consultations were performed directly between the physicians in the clinic and the patients, regardless of their location. No other medical providers, such as local caregivers or others, were involved. Patients received written instructions on how to conduct the video consultation, and no additional clinical staff were required to assist the patients. This pragmatic study design appeared to be the most promising one for a health economic evaluation seeking to produce valid, generalizable results [27]. Patients in the intervention group did not have to bear any additional costs or out-of-pocket payments for using telemedicine, as the digital health app or browser-based software was free for them to use. They were only required to have a smartphone, tablet, laptop, or computer with a microphone and camera, and an adequate internet connection. The examination itself was paid for by their respective health insurance. Patients in the control group did not have to pay any additional costs either; their costs for an in-clinic follow-up appointment (eg, travel costs) were the same as those they would have paid outside of study participation.

After the follow-up appointments, patients in both the intervention and control groups completed questionnaires. These questionnaires included questions about the distance between the patients’ homes and the clinic, the amount of time spent for the appointment (eg, travel and waiting time), and the potential need to be absent from work to attend the appointment. Further information on the study can be found in a previous publication by Muschol et al [13].

### Statistical Analysis and Health Economic Evaluation

The RCT data are presented as mean and SD, median and IQR, or percentage. To compare the intervention and control groups, the Mann-Whitney *U* test was used for continuous variables and the Fisher exact test was used for categorical ones. Statistical significance was assumed at *P*≤.05.

The health economic analysis was based on data collected from the questionnaires and other official, external data. The study design was guided by recommendations for health economic analyses in the context of eHealth interventions, and the study examined non–health care costs associated with the use of telemedicine from a societal perspective [27,28]. The analysis proceeded in two steps. In the first step, economic effects of the societal perspective were examined from the patients’ point of view. This involved, firstly, calculating and comparing three types of non–health care costs associated with medical appointments:

1. Travel costs were calculated following recommendations for empirical standard costs for health economic evaluations in Germany [29].
2. Time costs were assessed by assigning monetary values to patients’ travel time, waiting time, and total time spent on appointments based on Verbooy et al’s [30] valuation approach to unpaid work and leisure time.
3. Production losses due to patients’ absence from work while attending their appointments were computed using Germany’s average gross hourly wage in 2021 and average working hours for all German full-time and part-time employees in 2019 [31,32].

When tallying total costs from a societal perspective, it was felt to be appropriate to differentiate between patients who were employed and patients who were not employed, given that production losses are only relevant for patients who are employed.

In the second step, the effects of the societal perspective were evaluated in the form of the environmental impact of telemedicine. The analysis of the environmental impact was conducted using data from the German Federal Environment Agency. It comprised three different aspects. First, the environmental impact in terms of greenhouse gases, carbon monoxide, volatile hydrocarbons, nitrogen oxides, and particulates was calculated by multiplying the average emissions per passenger-kilometer (pkm) by the kilometers patients traveled by car to and from the clinic. This calculation was based on an average car occupancy of 1.4 passengers, as the average emissions are specified by the Federal Environment Agency on the basis of this value [33]. A separate calculation of emissions from public transportation was not performed within the study because only 1 patient in the control group and 1 patient in the telemedicine group used or would have used public transportation. Second, the average environmental costs incurred per pkm by the patients’ trips per car were calculated. For this purpose, the cost rate of the Federal Environment Agency of €195 per ton of carbon dioxide equivalent was applied (a currency exchange rate of €1=US $0.97 is applicable) [34,35]. This value is based on a higher weighting of the welfare of current versus future generations [35].

In a third step, the potential savings in emissions and environmental costs were estimated in a model calculation if 8 patients per week would conduct a video consultation instead of a clinic consultation, as was the case in our study [33-35].

### Sensitivity Analysis

Finally, a sensitivity analysis was performed to evaluate the robustness of the findings. For the patients’ point of view in the societal perspective, this analysis studied the effect of differentiating between full-time and part-time employment when calculating production losses [32]. For the environmental impact of the societal perspective, the sensitivity analysis considered the following:

1. A cost rate from the Federal Environment Agency for the calculation of the environmental costs of €680 per ton of carbon dioxide equivalent, which gives equal weight to the welfare of present and future generations [34,35].
2. A total of 16 patients with a video consultation per week for the analysis of potential savings in emissions and environmental costs [33-35].

For the calculation of the environmental costs, both €195 and €680 per ton of carbon dioxide equivalent were considered [34,35]. As the Federal Environment Agency reports both cost rates, the aim of the sensitivity analysis was to show how the equal weighting of the welfare of present and future generations (€680) compared to the higher weighting of the welfare of present versus future generations (€195) affects the environmental costs.

## Results

### General Findings

Of the 60 patients recruited—intervention group (n=30) and control group (n=30)—4 patients in each of the groups withdrew from the study. Thus, data from a total of 52 patients could be considered for the health economic evaluation, with several variables displaying a lower n value due to missing items on some patient questionnaires. The progress of the recruited patients through the trial is shown in a CONSORT flow diagram in Multimedia Appendix 1.

Demographic patient characteristics are shown in Table 1. No significant differences were observed between the telemedicine group and the control group.

Regarding the variables used for calculating costs, however, the differences between the groups were partially significant, as shown in Table 2. Treatment duration in the intervention group, at 8.23 minutes on average, was significantly shorter than that in the control group, at 10.92 minutes on average (*P*=.02). The average waiting time in the online waiting room for the telemedicine software was also significantly shorter than that experienced in the clinic (6.73 minutes vs 36.88 minutes, respectively; *P*<.001). The largest intergroup difference, however, was observed in total patient time spent per follow-up appointment. An appointment in the telemedicine group took an average of 21.92 minutes out of the patients’ days, whereas an appointment in the control group required patients to spend 154.80 minutes on average (*P*<.001). There was no significant difference between the potential travel distance and travel time the telemedicine group would have faced if required to travel to an in-clinic appointment and the actual travel distance and travel time faced by the control group. The groups also did not differ significantly in patients’ absence from work due to their appointments. Nevertheless, of the employed patients, only 5% (1/20) were absent from work so they could attend the appointment in the telemedicine group, compared with 16% (3/19) in the control group, as shown with the Fisher exact test (*P*=.34). In the telemedicine group, 1 patient had to visit the clinic again for further treatment. As this would also have been required after an F2F consultation and, therefore, occurred independently of the video consultation, this additional visit was not included in the cost calculation.

**Table 1:** Demographic characteristics of patients.

Characteristics		Telemedicine group (n=26), n (%)	Control group (n=26), n (%)	*P* value^a^	
**Medical indication**					.99
	Knee	10 (38)	9 (35)		
	Shoulder	16 (62)	17 (65)		
**Age (years)**					.36
	18-40	7 (27)	5 (19)		
	41-60	17 (65)	15 (58)		
	>60	2 (8)	6 (23)		
Female		11 (42)	10 (38)	.99	
Employed		20 (77)	19 (76)^b^	.99	

^a^*P* values were based on the Fisher exact test.

^b^Percentage of n=25 due to missing item on questionnaire.

**Table 2:** Variables included for cost calculation.

Variables	Telemedicine group (n=26)				Control group (n=26)				*P* value^a^		
	Participants, n (%)	Mean (SD)	Median (IQR)	Participants, n (%)		Mean (SD)	Median (IQR)				
Treatment duration (minutes)	26 (100)	8.23 (4.45)	6.00 (5-10)	25 (96)		10.92 (5.58)	10.00 (8-14.5)	.02			
Travel distance (kilometers)	26 (100)	37.00 (32.06)	30.00 (10-46.25)	25 (96)		31.58 (22.62)	28.00 (15.5-45)	.65			
Actual and potential travel time (minutes)	26 (100)	38.46 (21.72)	40.00 (18.75-46.25)	25 (96)		34.80 (20.89)	30.00 (20-40)	.42			
Waiting time (minutes)	26 (100)	6.73 (6.84)	5.00 (1.75-10)	24 (92)		36.88 (27.54)	30.00 (15-48.75)	<.001			
Total time spent on appointment (minutes)	26 (100)	21.92 (10.40)	22.50 (13.75-30)	25 (96)		154.80 (79.75)	150.00 (105-197.5)	<.001			

^a^*P* values were based on the Mann-Whitney *U* test.

### Patients’ Perspectives

The cost calculation from the patients’ point of view in the societal perspective showed that patients in the control group had to pay an average of €18.95 in travel costs, based on a cost of €0.30 for each kilometer travelled to and from the clinic, as shown in Table 3. There were no travel costs for patients in the telemedicine group because they did not have to attend the clinic. If they had had an in-clinic follow-up, however, their average travel costs would have been €22.20.

The time costs resulting from follow-up appointments in both groups were estimated at €16.00 per hour to account for both unpaid work time and leisure time that patients lost. The average cost of patients’ travel time was €18.56 in the control group. Again, patients in the telemedicine group faced no travel time costs due to the trip they avoided. Yet, the potential cost of their travel time would have been €20.51. The increased waiting time in the clinic was reflected in time costs of €9.83 in the control group, compared with €1.79 in the intervention group.

The difference in time costs between the groups became even more pronounced when the total time patients spent on their follow-up appointments was valued. Whereas patients with a telemedical appointment had average total time costs of €5.85, those with an in-clinic appointment had total time costs of €41.28. In other words, a telemedical rather than an in-clinic follow-up appointment would have saved patients €35.43 in average time costs.

Finally, the production loss due to patients’ absence from work while they were attending their appointments was calculated. This was based on an average hourly wage of €29.48 in Germany and an overall average of 6.96 working hours per day per full-time or part-time German employee. With 1 patient absent in the telemedicine group and 3 patients absent in the control group, total production losses were €205.18 and €615.54, respectively. With 20 employed patients in the telemedicine group and 19 employed patients in the control group, the costs due to lost production averaged €10.26 for a telemedical follow-up and €32.40 for an in-clinic one.

Taking employment status into account, the total costs of a follow-up appointment were €16.11 for an employed patient in the telemedicine group and €92.63 for an employed patient in the control group. For an unemployed patient, the total costs decreased to €5.85 in the telemedicine group and to €60.23 in the control group due to the irrelevant production loss. Multimedia Appendix 2 presents the cost calculations in detail.

**Table 3:** Cost calculation from the patients’ perspective.

Costs	Telemedicine group	Control group	Difference
Travel costs (€^a^), mean (SD)	0 (0)	18.95 (13.57)	18.95
Travel time costs (€), mean (SD)	0 (0)	18.56 (11.14)	18.56
Waiting time costs (€), mean (SD)	1.79 (1.82)	9.83 (7.34)	8.04
Total time costs (€), mean (SD)	5.85 (2.77)	41.28 (21.27)	35.43
Production loss (€)	205.18	615.54	410.36

^a^A currency exchange rate of €1=US $0.97 is applicable.

### Environmental Impact

To calculate the emissions saved in the telemedicine group due to the avoided trips to and from the clinic, 152 g/pkm for greenhouse gases, 0.94 g/pkm for carbon monoxide, 0.15 g/pkm for volatile hydrocarbons, 0.38 g/pkm for nitrogen oxides, and 0.006 g/pkm for particulates were applied based on an average car occupancy of 1.4 passengers. This led to the result that around 11.248 kg of greenhouse gases, 0.070 kg of carbon monoxide, 0.011 kg of volatile hydrocarbons, 0.028 kg of nitrogen oxides, and 0.0004 kg of particulates were saved per patient with the help of video consultations. Table 4 also shows the total emissions saved for the 26 patients in the telemedicine group. For example, as a result of the video consultations, emissions of 292.448 kg of greenhouse gases could be avoided in our study. The calculation of environmental costs saved in the telemedicine group is based on environmental costs of €0.05045 per pkm. This value represents the average environmental costs of gasoline and diesel powered cars. The use of telemedicine saved approximately €3.73 in environmental costs per patient, resulting in a total of €97.07 for all patients in our study. Finally, the potential savings can also be seen in the model calculation for 1 year if 8 patients per week had a video consultation instead of a clinic consultation, as was the case in our study. For this calculation, the average distance between the home of the patients in the telemedicine group and control group and the clinic was used. With a total of 384 patients who would not have to travel to the clinic each year due to video consultations, a total of 4009.88 kg of greenhouse gases, 24.80 kg of carbon monoxide, 3.96 kg of volatile hydrocarbons, 10.02 kg of nitrogen oxides, and 0.16 kg of particulates could be avoided. In addition, at €195 per ton of carbon dioxide equivalent, €1330.91 in environmental costs could be saved.

**Table 4:** Saved emissions and environmental costs in the telemedicine group.

Emissions and costs	Per patient	Total
Greenhouse gases (kg)	11.248	292.448
Carbon monoxide (kg)	0.070	1.809
Volatile hydrocarbons (kg)	0.011	0.289
Nitrogen oxides (kg)	0.028	0.731
Particulates (kg)	0.0004	0.012
Environmental costs (€^a^)	3.73	97.07

^a^A currency exchange rate of €1=US $0.97 is applicable.

### Sensitivity Analysis

In the subsequent sensitivity analysis, several adjustments were made. First, the cost calculation from the patients’ point of view was modified to test the effect of alternative assumptions on the valuation of production losses. Assuming that all patients who were absent from work were employed full time (ie, 8.2 hours per day), the societal cost of lost production would have increased to €241.74 (mean €12.09, SD 54.05) in the telemedicine group and to €725.21 (mean €38.17, SD 90.56) in the control group. In contrast, assuming only part-time employment of 3.9 hours per day for all patients who were absent from work, the costs of lost production would have decreased to €114.97 (mean €5.75, SD 25.71) in the telemedicine group and to €344.92 (mean €18.15, SD 43.07) in the control group. These assumptions would have changed the total costs for employed patients to €17.94 for full-time employees and €11.60 for part-time employees in the telemedicine group, as well as to €98.40 for full-time employees and €78.38 for part-time employees in the control group.

Second, the calculation of environmental costs was adjusted to the cost rate of €680 per ton of carbon dioxide equivalent, which increased the average environmental costs of gasoline and diesel cars to €0.12885 per pkm. Due to this adjustment, the environmental costs saved in the telemedicine group would have been €9.53 per patient and €247.91 in total.

In addition, if a total of 16 patients per week had a video consultation instead of a clinic consultation, approximate emissions of 8019.76 kg of greenhouse gases, 49.60 kg of carbon monoxide, 7.91 kg of volatile hydrocarbons, 20.05 kg of nitrogen oxides, and 0.32 kg of particulates could be saved. Environmental costs could furthermore be reduced by €2661.82, at €195 per ton of carbon dioxide equivalent, or by €6798.33, at €680 per ton of carbon dioxide equivalent.

## Discussion

### Principal Findings

This analysis of the economics of using telemedicine in follow-up care for patients in orthopedic and trauma surgery in a German university hospital showed that implementing video consultations enabled time and cost savings for patients, savings in environmental costs, and reductions in emissions.

### Implications for Patients

Seen from the patients’ point of view in the societal perspective of the health economic analysis, the use of telemedicine was not associated with additional costs (eg, out-of-pocket payments) for the patients in our study. On the contrary, compared with the control group, telemedical appointments resulted in cost savings due to the avoidance of travel and the reduction in time costs.

Previous economic evaluations by Buvik et al [36] and Ohinmaa et al [37] also showed that telemedicine saved travel time and travel distance—and, thus, travel costs—in sparsely populated Scandinavian countries even though patients had to travel to a local caregiver for their appointment [36,37]. Similarly, RCTs by Sathiyakumar et al [9] and Kane et al [12] found savings in travel distances and time spent as well, but these studies did not feature economic analyses [9,12]. Reducing travel burdens is an important societal benefit of telemedicine, as it can ensure better access to medical care. In particular, patients in rural regions and hospitals that seek to offer their medical services beyond their own region stand to benefit. At the same time, however, all patients must still be able to reach their local clinic when video consultations are not sufficient.

Since our trial ended in 2021, our analysis did not consider the energy pricing dynamics following the 2022 European energy crisis. Actual savings in travel costs could be far higher in future digital health deployments.

In addition, the results of the analysis showed that the average costs of lost production were lower for a video consultation compared to a clinical consultation, indicating that telemedicine may have a positive impact in this regard as well. The potential of telemedicine to reduce lost work time—and, thus, production losses—reported here is consistent with the findings of other RCTs [9,12,36,37].

From a societal point of view, the use of telemedicine saved average total costs for employed patients of €76.52 per follow-up appointment, ranging from €66.78 to €80.46 in the sensitivity analysis. Most likely, the real savings would be even higher, as patients often wish or require an accompanying person for a clinic consultation, and the cost and time savings of companions were not considered in the study. The finding that video consultations save overall costs compared with conventional F2F examinations in follow-up care is also confirmed by Buvik et al’s [36] analysis. It should be noted, however, that in our calculation patient time lost due to a follow-up appointment was assigned a monetary value independently of any production losses, because including such time costs is strongly recommended in health economic methodology [28,30].

### Implications for the Environment

In addition, from the environmental point of view in the societal perspective, our analysis showed that for each patient who received a video consultation instead of a clinic consultation, emissions of 11.248 kg of greenhouse gases, 0.070 kg of carbon monoxide, 0.011 kg of volatile hydrocarbons, 0.028 kg of nitrogen oxides, and 0.0004 kg of particulates could be saved due to avoiding traveling by car. International studies have also demonstrated the reduction of emissions through the use of telemedicine, although the level of individual emissions differs in the respective studies [38,39]. For example, in a study by Udayaraj et al [23], telemedicine led to a reduction of 3527 miles and saved 1035 kg of carbon dioxide for kidney transplant patients in the United Kingdom. A retrospective analysis of patients in vascular surgery in the United States by Paquette and Lin [24] found a reduction of 1632 kg of carbon dioxide; 42,867 g of carbon monoxide; and 3160 g of nitric oxides by performing a total of 146 telemedicine encounters. In addition, based on Spanish data, a study by Vidal-Alaball et al [25] showed an average reduction of 3248.3 g of carbon dioxide, 4.05 g of carbon monoxide, and 4.86 g of nitric oxides per patient in a telemedicine program that included different specialties.

In our study, up to 8 patients could be treated weekly via telemedicine, which can lead to an annual improvement in the environmental footprint for a single German university orthopedic and trauma surgery department alone. Although the performance of telemedicine is not suitable for all patients in orthopedic and trauma surgery, the reduction in emissions could be improved by increasing the number of patients treated by video consultations each week. If the number of patients were expanded to the 1903 hospitals in Germany and included specialties suitable for telemedicine, such as general and visceral surgeries or dermatology, the call of the 125th German Medical Assembly in 2021 for a net-zero German health care system could be substantially supported [22].

In addition to the emission savings themselves, our study also showed that the introduction of telemedicine can also contribute to a reduction in environmental costs from the societal perspective.

### Implications for Practice

This health economic analysis provides clinical evidence that can improve stakeholders’ decision-making on implementing telemedicine both in and beyond the current COVID-19 pandemic. It was shown that the use of telemedicine in the follow-up care of orthopedic and trauma surgery benefits both patients and the environment from an economic perspective. Given the pragmatic design of this study, it can be expected that its main findings can be applied by decision makers in other clinical contexts as well.

When deciding whether to implement telemedicine, however, health care providers should consider other aspects besides the economic and environmental benefits. First, the quality of care provided by telemedicine must be ensured. Patient and physician satisfaction, efficiency, and the safety of the video consultations in terms of the same clinical outcomes achieved in F2F consultations play an important role. Various studies show that these goals can be achieved by introducing telemedicine in orthopedic and trauma surgery [6-12]. In addition, we have extensively analyzed patient and physician satisfaction, as well as quality of care for the study cohort in a previous publication [13]. Second, the costs of the technological infrastructure for telemedicine (eg, for electricity, internet connection, and hardware, such as computers and laptops with cameras and microphones) have to be considered. This infrastructure, however, is expected to be part of the standard equipment in most hospitals, as was the case in our study.

### Limitations

This study also has some limitations that should be noted. First, although the results were primarily based on actual data collected in the course of an RCT, some assumptions had to be made to be able to calculate costs. Travel costs saved, for example, were calculated based on the assumption that patients have their video consultations at home. In fact, they could have them anywhere, meaning that patients’ actual travel costs from that place to the hospital may well be higher or lower. The distance from home and the time spent on the appointments (eg, travel and waiting times) were furthermore queried via a questionnaire, and the actual distances and times could potentially differ slightly from the information provided by the patients. In addition, the original calculation of production loss lacked information on whether patients were employed full time or part time. For this reason, a sensitivity analysis sought to identify possible deviations and to evaluate the robustness of the findings.

Furthermore, given that data on time costs for German patients were missing in the literature, Verbooy et al’s [30] valuation approach was used, which was based on Dutch data. However, assuming that the Dutch population is reasonably similar to the German one, this minor inconsistency appears unlikely to have distorted overall results.

Finally, one of the inclusion criteria of the study was patients’ ownership of a technical device (smartphone, computer, etc) that allowed them to make video calls. This requirement could lead to socioeconomic inequalities being exacerbated, because only patients with adequate financial means might be able to benefit from cost savings due to telemedicine [40]. This inequity could not be avoided within the study, but it is an important issue with practical relevance and should be taken into account by policy makers.

### Conclusions

The use of telemedicine was found to reduce the environmental footprint and to save travel costs, travel time, and time costs for patients, and it helped to lower production losses from a societal perspective compared to F2F consultations in Germany. Thus, telemedicine helps to reduce costs in multiple dimensions. These results were demonstrated in the first health economic analysis of the use of telemedicine in follow-up care for patients with knee and shoulder disorders in orthopedic and trauma surgery, based on data from Germany. Simultaneously, this study provided economic and environmental evidence supporting stakeholders, such as hospitals, patients, and policy makers, who may consider extending the use of telemedicine in and beyond the COVID-19 pandemic. In addition, these findings might be relevant beyond the medical specialty of orthopedic and trauma surgery; they could be applied to other clinical contexts and to a wide range of potential digital health applications that substitute outpatient hospital visits with video consultations.

## Appendix

Multimedia Appendix 1 Consolidated Standards of Reporting Trials (CONSORT) flow diagram.

Multimedia Appendix 2 Detailed presentation of cost calculations.

Multimedia Appendix 3 CONSORT-eHEALTH checklist (V 1.6.1).

## Abbreviations

CONSORT: Consolidated Standards of Reporting Trials
F2F: face-to-face
pkm: passenger-kilometer
RCT: randomized controlled trial

## Data Availability

The data sets generated and analyzed during this study are available from the corresponding author upon reasonable request.

## References

[1] Wood G, Naudie D, MacDonald S, McCalden R, Bourne R (2011). An electronic clinic for arthroplasty follow-up: A pilot study. Can J Surg.

[2] Lanham N, Bockelman K, McCriskin B (2020). Telemedicine and orthopaedic surgery. JBJS Rev.

[3] Parisien RL, Shin M, Constant M, Saltzman BM, Li X, Levine WN, Trofa DP (2020). Telehealth utilization in response to the novel coronavirus (COVID-19) pandemic in orthopaedic surgery. J Am Acad Orthop Surg.

[4] Jaenisch M, Kohlhof H, Touet A, Kehrer M, Cucchi D, Burger C, Wirtz DC, Welle K, Kabir K (2021). Evaluation of the feasibility of a telemedical examination of the hip and pelvis - Early lessons from the COVID-19 pandemic. Z Orthop Unfall.

[5] Kolin DA, Carroll KM, Plancher K, Cushner F (2021). Perspective of attending physicians on the use of telemedicine in an outpatient arthroplasty setting during the COVID-19 pandemic. HSS J.

[6] Vuolio S, Winblad I, Ohinmaa A, Haukipuro K (2003). Videoconferencing for orthopaedic outpatients: One-year follow-up. J Telemed Telecare.

[7] Good DW, Lui DF, Leonard M, Morris S, McElwain JP (2012). Skype: A tool for functional assessment in orthopaedic research. J Telemed Telecare.

[8] Sharareh B, Schwarzkopf R (2014). Effectiveness of telemedical applications in postoperative follow-up after total joint arthroplasty. J Arthroplasty.

[9] Sathiyakumar V, Apfeld J, Obremskey W, Thakore RV, Sethi MK (2015). Prospective randomized controlled trial using telemedicine for follow-ups in an orthopedic trauma population. J Orthop Trauma.

[10] Buvik A, Bugge E, Knutsen G, Småbrekke A, Wilsgaard T (2016). Quality of care for remote orthopaedic consultations using telemedicine: A randomised controlled trial. BMC Health Serv Res.

[11] Buvik A, Bugge E, Knutsen G, Småbrekke A, Wilsgaard T (2018). Patient reported outcomes with remote orthopaedic consultations by telemedicine: A randomised controlled trial. J Telemed Telecare.

[12] Kane LT, Thakar O, Jamgochian G, Lazarus MD, Abboud JA, Namdari S, Horneff JG (2020). The role of telehealth as a platform for postoperative visits following rotator cuff repair: A prospective, randomized controlled trial. J Shoulder Elbow Surg.

[13] Muschol J, Heinrich M, Heiss C, Knapp G, Repp H, Schneider H, Thormann U, Uhlar J, Unzeitig K, Gissel C (2022). Assessing telemedicine efficiency in follow-up care with video consultations for patients in orthopedic and trauma surgery in Germany: Randomized controlled trial. J Med Internet Res.

[14] Al Knawy B, Adil M, Crooks G, Rhee K, Bates D, Jokhdar H, Klag M, Lee U, Mokdad AH, Schaper L, Al Hazme R, Al Khathaami AM, Abduljawad J (2020). The Riyadh Declaration: The role of digital health in fighting pandemics. Lancet.

[15] Petersen W, Karpinski K, Backhaus L, Bierke S, Häner M (2021). A systematic review about telemedicine in orthopedics. Arch Orthop Trauma Surg.

[16] Foni NO, Costa LAV, Velloso LMR, Pedrotti CHS (2020). Telemedicine: Is it a tool for orthopedics?. Curr Rev Musculoskelet Med.

[17] Moisan P, Barimani B, Antoniou J (2021). Orthopedic surgery and telemedicine in times of COVID-19 and beyond: A review. Curr Rev Musculoskelet Med.

[18] Haider Z, Aweid B, Subramanian P, Iranpour F (2022). Telemedicine in orthopaedics during COVID-19 and beyond: A systematic review. J Telemed Telecare.

[19] McMaster T, Wright T, Mori K, Stelmach W, To H (2021). Current and future use of telemedicine in surgical clinics during and beyond COVID-19: A narrative review. Ann Med Surg (Lond).

[20] Cole PA, Lezak BA, Schroder LK, Cole PA (2021). Global orthopaedic trauma surgeons highlight telenomics during the COVID-19 era: A case for advancing telemedicine in orthopaedics. J Clin Orthop Trauma.

[21] Oates EV, Lim GHC, Nevins EJ, Kanakala V (2021). Are surgical patients satisfied with remote consultations? A comparison of remote versus conventional outpatient clinic follow-up for surgical patients: A systematic review and meta-analysis of randomized controlled trials. J Patient Exp.

[22] Gießelmann K, Osterloh F (2021). Deutscher Ärztetag. Klimaschutz im Gesundheitswesen: Klimaneutralität bis 2030 [Article in German]. aerzteblatt.de.

[23] Udayaraj UP, Watson O, Ben-Shlomo Y, Langdon M, Anderson K, Power A, Dudley C, Evans D, Burhouse A (2019). Establishing a tele-clinic service for kidney transplant recipients through a patient-codesigned quality improvement project. BMJ Open Qual.

[24] Paquette S, Lin JC (2019). Outpatient telemedicine program in vascular surgery reduces patient travel time, cost, and environmental pollutant emissions. Ann Vasc Surg.

[25] Vidal-Alaball J, Franch-Parella J, Lopez Seguí F, Garcia Cuyàs F, Mendioroz Peña J (2019). Impact of a telemedicine program on the reduction in the emission of atmospheric pollutants and journeys by road. Int J Environ Res Public Health.

[26] Moher D, Hopewell S, Schulz KF, Montori V, Gøtzsche PC, Devereaux PJ, Elbourne D, Egger M, Altman DG (2010). CONSORT 2010 explanation and elaboration: Updated guidelines for reporting parallel group randomised trials. BMJ.

[27] Bergmo TS (2015). How to measure costs and benefits of eHealth interventions: An overview of methods and frameworks. J Med Internet Res.

[28] Sanders GD, Neumann PJ, Basu A, Brock DW, Feeny D, Krahn M, Kuntz KM, Meltzer DO, Owens DK, Prosser LA, Salomon JA, Sculpher MJ, Trikalinos TA, Russell LB, Siegel JE, Ganiats TG (2016). Recommendations for conduct, methodological practices, and reporting of cost-effectiveness analyses: Second panel on cost-effectiveness in health and medicine. JAMA.

[29] Krauth C, Hessel F, Hansmeier T, Wasem J, Seitz R, Schweikert B (2005). [Empirical standard costs for health economic evaluation in Germany -- A proposal by the working group methods in health economic evaluation] [Article in German]. Gesundheitswesen.

[30] Verbooy K, Hoefman R, van Exel J, Brouwer W (2018). Time is money: Investigating the value of leisure time and unpaid work. Value Health.

[31] (2022). National accounts, domestic product. German Federal Statistical Office (Destatis).

[32] (2021). Quality of employment - Weekly working hours. German Federal Statistical Office (Destatis).

[33] (2022). Emissionsdaten. Umweltbundesamt [Content in German].

[34] (2021). Gesellschaftliche Kosten von Umweltbelastungen. Umweltbundesamt [Content in German].

[35] Matthey A, Bünger B (2020). Methodenkonvention 3.1 zur Ermittlung von Umweltkosten: Kostensätze. Umweltbundesamt [Content in German].

[36] Buvik A, Bergmo TS, Bugge E, Smaabrekke A, Wilsgaard T, Olsen JA (2019). Cost-effectiveness of telemedicine in remote orthopedic consultations: Randomized controlled trial. J Med Internet Res.

[37] Ohinmaa A, Vuolio S, Haukipuro K, Winblad I (2002). A cost-minimization analysis of orthopaedic consultations using videoconferencing in comparison with conventional consulting. J Telemed Telecare.

[38] Purohit A, Smith J, Hibble A (2021). Does telemedicine reduce the carbon footprint of healthcare? A systematic review. Future Healthc J.

[39] Ravindrane R, Patel J (2022). The environmental impacts of telemedicine in place of face-to-face patient care: A systematic review. Future Healthc J.

[40] OECD (2021). Health at a Glance 2021: OECD Indicators.

