# Supplementary material for: Economic and Environmental Impact of Digital Health App Video Consultations in Follow-up Care for Patients in Orthopedic and Trauma Surgery in Germany: Randomized Controlled Trial
Source: J Med Internet Res. 2022 Nov 24;24(11):e42839. doi: 10.2196/42839 (PMC9732751; doi:10.2196/42839)
Supplement: Multimedia Appendix 1 [file jmir_v24i11e42839_app1.docx]

**Multimedia Appendix 1:**

**CONSORT (Consolidated Standards of Reporting Trials) flow diagram**
