# Supplementary material for: Economic and Environmental Impact of Digital Health App Video Consultations in Follow-up Care for Patients in Orthopedic and Trauma Surgery in Germany: Randomized Controlled Trial
Source: J Med Internet Res. 2022 Nov 24;24(11):e42839. doi: 10.2196/42839 (PMC9732751; doi:10.2196/42839)
Supplement: Multimedia Appendix 2 [file jmir_v24i11e42839_app2.docx]

**Multimedia Appendix 2: Detailed presentation of cost calculations**

| Table S1: Detailed presentation of cost calculation from the patients’ point of view | | | | |
| --- | --- | --- | --- | --- |
|  | **Telemedicine group** | | **Control group** | |
|  | Calculation | (Mean) Costs | Calculation | (Mean) Costs |
| Average travel costs | 0  (Potential: 37.00 km x 2 x 0.3) | €0  (Potential: €22.20) | 31.58 km x 2 x 0.3 | €18.95 |
| Average travel time costs | 0  (Potential: 38.46 minutes x 2 x  (€16/60 minutes)) | 0  (Potential: €20.51) | 34.8 minutes x 2 x (€16/60 minutes) | €18.56 |
| Average waiting time costs | 6.73 minutes x (€16/60 minutes) | €1.79 | 36.88 minutes x (€16/60 minutes) | €9.83 |
| Average total  time costs | 21.92 minutes x (€16/60 minutes) | €5.85 | 154.8 minutes x (€16/60 minutes) | €41.28 |
| Production loss | ((34.8 hours/5 days) x €29.48 x 1 patient)/20 patients | Total: €205.18  Mean: €10.26 | ((34.8 hours/5 days) x €29.48 x 3 patient)/19 patients | Total: €615.54  Mean: €32.40 |
| Total costs | Patients in employment:  Travel costs + Total time costs + Production loss  Patients not in employment:  Travel costs + Total time costs | Patients in employment: €16.11  Patients not in employment: €5.85 | Patients in employment:  Travel costs + Total time costs + Production loss  Patients not in employment:  Travel costs + Total time costs | Patients in employment: €92.63  Patients not in employment: €60.23 |

| Table S2: Detailed presentation of cost calculation of the environmental impact | | | | |
| --- | --- | --- | --- | --- |
|  | **Per patient** | | **In total** | |
|  | Calculation | Emissions/costs | Calculation | Emissions/costs |
| Greenhouse gases | (37.0 km x 2 x 152 g/pkm)/ 1000 | 11.248 kg | ((37.0 km x 2 x 152 g/pkm)/ 1000)  x 26 | 292.448 kg |
| Carbon monoxide | (37.0 km x 2 x 0.94 g/pkm)/ 1000 | 0.070 kg | ((37.0 km x 2 x 0.94 g/pkm)/ 1000) x 26 | 1.809 kg |
| Volatile hydrocarbons | (37.0 km x 2 x 0.15 g/pkm)/ 1000 | 0.011 kg | ((37.0 km x 2 x 0.15 g/pkm)/ 1000) x 26 | 0.289 kg |
| Nitrogen oxides | (37.0 km x 2 x 0.38 g/pkm)/ 1000 | 0.028 kg | ((37.0 km x 2 x 0.38 g/pkm)/ 1000) x 26 | 0.731 kg |
| Particulates | (37.0 km x 2 x 0.006 g/pkm)/ 1000 | 0.0004 kg | ((37.0 km x 2 x 0.006 g/pkm)/ 1000) x 26 | 0.012 kg |
| Environmental costs per €195 per ton of carbon dioxide equivalent | 37.0 km x 2 x €0.05045/pkm | €3.73 | (37.0 km x 2 x €0.05045/pkm) x 26 | €97.07 |
| Environmental costs per €680 per ton of carbon dioxide equivalent | 37.0 km x 2 x €0.12885/pkm | €9.53 | (37.0 km x 2 x €0.12885/pkm) x 26 | €247.91 |
